# *Clostridium difficile* PCR Ribotypes in Calves, Canada

**DOI:** 10.3201/eid1211.051581

**Published:** 2006-11

**Authors:** Alexander Rodriguez-Palacios, Henry R. Stämpfli, Todd Duffield, Andrew S. Peregrine, Lise A. Trotz-Williams, Luis G. Arroyo, Jon S. Brazier, J. Scott Weese

**Affiliations:** * University of Guelph, Guelph, Ontario, Canada;; † University Hospital of Wales, Cardiff, United Kingdom3

**Keywords:** Calf diarrhea, Clostridium difficile, humans, Cryptosporidium, cattle, fluoroquinolones, zoonosis, levofloxacin, tcdC, antimicrobial drug resistance, research

## Abstract

We investigated *Clostridium difficile* in calves and the similarity between bovine and human *C. difficile* PCR ribotypes by conducting a case-control study of calves from 102 dairy farms in Canada. Fecal samples from 144 calves with diarrhea and 134 control calves were cultured for *C. difficile* and tested with an ELISA for *C. difficile* toxins A and B. *C. difficile* was isolated from 31 of 278 calves: 11 (7.6%) of 144 with diarrhea and 20 (14.9%) of 134 controls (p = 0.009). Toxins were detected in calf feces from 58 (56.8%) of 102 farms, 57 (39.6%) of 144 calves with diarrhea, and 28 (20.9%) of 134 controls (p = 0.0002). PCR ribotyping of 31 isolates showed 8 distinct patterns; 7 have been identified in humans, 2 of which have been associated with outbreaks of severe disease (PCR types 017 and 027). *C*. *difficile* may be associated with calf diarrhea, and cattle may be reservoirs of *C. difficile* for humans.

## Abstract 2

*C*. *difficile*, including epidemic PCR ribotypes 017 and 027, were isolated from dairy calves in Canada.

Clostridium difficile, a gram-positive, spore-forming, anaerobic bacterium, has been associated with pseudomembranous colitis and nosocomial and antimicrobial drug–associated diarrhea in humans (*1*). Recently, research has suggested that the frequency, severity, and relapse of C. difficile–associated disease (CDAD) are increasing in Europe and North America (*1**,**2*). The most common risk factor for CDAD in humans is the use of antimicrobial drugs, particularly fluoroquinolones (*3**–**5*). Of recent concern, hypervirulent C. difficile strains have been associated with outbreaks of severe CDAD (*2**,**6*).

The pathophysiology of CDAD involves colonization of the intestinal tract with C. difficile and production of its toxins (*7**–**9*). At least 3 cytotoxins are currently described for C. difficile: toxins A and B (glucosyltransfersases) and a binary toxin (CDT, ADP-ribosyltransferase) (*10*). Toxins TcdA and TcdB are encoded by 2 separate genes, tcdA and tcdB, located in a 19.6-kb pathogenicity locus (PaLoc). The expression of these 2 genes is regulated by a putative negative regulator within PaLoc, the tcdC gene (*11*). Deletions in tcdC are believed to result in overexpression of tcdA and tcdB and increased production of toxins A and B, which may account for the apparent higher pathogenicity in certain ribotypes (i.e., PCR type 027) (*1*). Some strains also produce binary toxin, which is encoded by the genes cdtA and cdtB located outside PaLoc (*10*). The role of binary toxin in disease is currently under investigation (*12*). Isolates producing >1 of these toxins (A, B, or binary) are currently referred to as toxigenic strains (*10*). C. difficile also appears to be an important cause of enteric disease in other species, including horses, dogs, and pigs (*7**,**8**,**13**,**14*).

Neonatal calf diarrhea (NCD) is a common cause of illness (10.2%) and death in preweaning calves (*15*). A variety of enteropathogens have been implicated in NCD; however, many cases are currently idiopathic (*16*). Although C. difficile infection has been suggested as a cause of diarrhea and enteritis in calves (*17*), further published evidence is lacking. The objectives of this study were to evaluate the role of C. difficile in NCD, genotypically and phenotypically characterize isolates from calves, and compare calf and human isolates.

## Materials and Methods

### Farms and Calves

A total of 102 dairy farms in southern Ontario, Canada, were included in the study. Farms were visited from May through September 2004 to obtain 1 fecal sample from calves <1 month of age. Fecal samples (>4 g) were obtained from 10 consecutively born calves per farm. Samples were scored at the farm using a 4-grade fecal scoring system and then stored at 4°C within 6 hours of collection. A score of 1 represented hard, dry fecal matter; score 2, pasty and sticky feces; score 3 soft feces; and score 4, watery feces that would adopt the shape of the container immediately after sampling. Samples with a score of 4 were considered to have diarrhea, whereas scores of 1 and 2 were controls. Samples with a score of 3 were discarded to reduce selection bias. Selected samples were recoded for blinding purposes and stored at –70°C within 24 hours of collection. A questionnaire that requested information about colostrum quality and administration, diet, housing, cleaning and disinfection practices, antimicrobial or antiprotozoal feed supplements, level of nose-to-nose contact among calves, vaccination of dams, and dehorning was administered on each farm to investigate risk factors for C. difficile in feces.

### C. difficile Culture and Detection of Toxins A and B

Fecal samples were processed within 2 hours after thawing. Enrichment culture was performed as previously described (*7**,**18*). Briefly, ≈1 g of homogenized fecal matter was mixed with 2 mL of 96% ethanol and agitated at room temperature for 50 minutes to select for bacterial spores. The sediment was recovered after centrifugation at 3,800 × g for 10 minutes and resuspended in 5 mL of cycloserine-cefoxitin fructose broth (C. difficile agar and C difficile supplement SR0096; Oxoid, Columbia, MD, USA) that was incubated anaerobically at 37°C for 7 days. This broth was treated with 96% ethanol (1:1 vol/vol), centrifuged at 3,800 × g for 10 minutes, and the sediment was resuspended in 200 μL of sterile deionized water. Thereafter, 200 μL of sediment was streaked onto cycloserine-cefoxitin fructose agar and blood agar that were incubated anaerobically at 37°C. Plates were evaluated in an anaerobic environment daily for <5 days. If present, at least 2 colonies (swarming, flat, rough, nonhemolytic) were subcultured. C. difficile was identified by Gram stain (spore-forming gram-positive rods) and detection of L-proline aminopeptidase activity (Pro Disc, Remel, Lenexa, KS, USA) (*19*). Isolates were stored at –70°C until molecular analyses were performed.

Feces were screened for C. difficile toxins A and B by using an ELISA (Tox A/B ELISA, TechLab, Blacksburg, VA, USA) (*20*). The test was performed per manufacturer's instructions. Two observers interpreted the reactions in a blinded fashion.

### Extraction of DNA

DNA was extracted by using a Chelex resin-based kit (InstaGene Matrix, Bio-Rad, Laboratories, Hercules, CA, USA) (*21*). After centrifugation of the C. difficile DNA–containing solutions, 125 μL of supernatant was collected and stored at –20°C as a template for PCR analyses.

### PCR Ribotyping

PCR ribotyping analyses were performed as previously described (*22*). DNA was amplified by using a thermal cycler (Touchgene Gradient, Techne Inc., Burlington, NJ, USA). Ribotype patterns were compared visually with C. difficile PCR ribotypes from humans and other animals from the provinces of Ontario, Quebec, and Manitoba, Canada. The first isolate identified for each PCR ribotype was submitted to the Anaerobe Reference Laboratory, University Hospital of Wales, Cardiff, United Kingdom, for comparison (i>23).

### Detection of tcdA, tcdB, tcdC, and cdtB Genes

Amplification of nonrepeating and repeating sequences of the tcdA gene and the nonrepeating sequences of the tcdB gene was performed as previously described (*24*). Identification of tcdC and cdtB genes was based on previous protocols (*11**,**24**,**25*). Reference strains were included as positive and negative controls in every experiment.

### Antimicrobial Drug Susceptibility Tests

MICs for metronidazole, clindamycin, levofloxacin, and vancomycin were determined by using the E-test method (AB Biodisk, Solna, Sweden) (*26*). A McFarland standard 1 suspension of pure C. difficile colonies was placed on Muller-Hinton blood agar plates (Oxoid, Basingstoke, UK). After 48 hours of anaerobic incubation, MICs were determined by consensus of 2 observers.

### Toxinotyping of C. difficile Strains

Toxinotyping analysis involved amplification and enzymatic restriction of PCR fragment A3 of tcdA and PCR fragment B1 of tcdB. This was performed following a previously published protocol (*27*).

### Other Enteropathogens

Because intestinal cryptosporidiosis was common (40.6%) in dairy calves <28 days of age in the study area (*16*), samples examined for C. difficile were also tested for Cryptosporidium spp. oocysts (sucrose wet mount test) to control for potential interactions regarding diarrhea. Other calf enteropathogens were not investigated because they are less prevalent in the region (L.A. Trotz-Williams et al., unpub. data).

### Statistical Analysis

Multivariate stepwise logistic regression analyses were performed by using SAS statistical software (SAS Institute, Cary, NC, USA). Associations between farm management data, age, sampling month, and results from laboratory tests were investigated by using a generalized model procedure (GenMod in SAS). Variables associated with diarrhea and C. difficile or its toxins in feces were investigated. During initial model building, variables with p<0.15 were selected to construct final models. Parameters were considered statistically significant if p values were <0.05. A generalized linear mixed model controlling for farm as a random effect was used to estimate and test the farm variance component. Relationships between C. difficile toxins and diarrhea and between C. difficile toxins and the age and month of sampling were investigated in the models. Pairwise comparisons of least square means were performed, and approximated Tukey adjusted p values were computed. Reported exact p values, odd ratios (ORs), and 95% confidence intervals (CIs) were determined with exact conditional logistic regression tests by using LogXact 5 software (Cytel Inc., Cambridge, MA, USA) when analyses did not yield exact values with SAS software.

## Results

A total of 278 calves were studied: 144 with diarrhea and 134 controls. The mean age of the sample was 14.2 days (range 5–30 days); 39 calves were 5–7 days of age, 107 were 8–14 days of age, 96 were 15–21 days of age, and 32 were 22–30 days of age. Four calves had no age recorded and were not used for descriptive information regarding age. The mean ages of the control calves (14.8 days, 95% CI 13.7–15.9) and calves with diarrhea (13.9 days, 95% CI 13.0–14.7) were not significantly different (p = 0.16).

C. difficile was isolated from 31 (11.2%) of 278 calves from 25 (25%) of 102 farms. This bacterium was more commonly identified in feces from control calves (14.9%, 20/134) than in feces from calves with diarrhea (7.6%, 11/144) (OR 3.47, 95% CI 1.27–10.24, exact 2-tailed p = 0.009).

C. difficile toxins A and B were identified in feces of 85 (30.6%) of 278 calves from 58 (56.8%) of 102 farms: 57 (39.6%) of 144 calves with diarrhea and 28 (20.9%) of 134 controls (OR 3.07, 95% CI 1.62–5.96, exact 2-tailed p = 0.0002). C. difficile and its toxins were detected concurrently in only 6 (4.2%) of 144 calves with diarrhea and in 7 (5.2%) of 134 controls.

Generalized linear mixed model analysis with farm as a random effect showed no farm variance component (coefficient 0). Thus, farm was included in subsequent models as a fixed effect. Generalized linear model analyses showed that none of the farm management practices surveyed were associated with diarrhea or C. difficile test results. Conversely, the month of sampling (p = 0.008) and the age of the calves (p = 0.005) were significant variables when modeling for the ELISA result as the outcome. May, June, and July were associated with higher ORs of yielding a positive fecal C. difficile toxin test result than was August (Table 1).

**Table 1:** Estimated odds ratios for a calf to have a positive toxin A/B ELISA result, southern Ontario, Canada, 2004*

Pairwise comparison	Odds ratio	95% CI	Adjusted Tukey p values

May vs August†	3.62	1.8–8.3	0.007
June vs August†	3.17	1.3–7.7	0.029
July vs August†	2.58	1.2–5.5	0.038
May vs July	1.41	0.7–2.8	0.59
June vs July	1.23	0.6–2.6	0.85
May vs June	1.14	0.5–2.6	0.95


*CI, confidence interval. The number of positive toxin A/B ELISA test results and calves sampled per month was May, 23/57; June, 16/41; July, 34/105; and August, 12/75. August data include 2 observations from September.†Statistically significant.

When the association with age was analyzed, a linear relationship was found between age of calves and probability of a positive test result for C. difficile toxins. Fecal samples from older calves were less likely than samples from younger calves to be positive for C. difficile toxins; the estimated OR was 2.0 for every 10 days of age difference at any point from 5 and 30 days of age (natural antilogarithm of [0.0691 × no. of days of interest]; 95% CI 1.22–3.24, p = 0.006). No association was found between administration of feed supplemented with oxytetracycline (33 calves on 11 farms) or anticoccidial drugs (251 calves on 91 farms) and C. difficile and its toxins in feces.

Cryptosporidium spp. oocysts in feces were significantly associated with diarrhea and identified in 80 (55.9%) of 144 calves with diarrhea and 19 (14.2%) of 134 control calves (OR 8.23, 95% CI 4.35–16.26, exact 2-tailed p<0.0001). However, generalized linear model analysis showed no interaction between Cryptosporidium spp. and C. difficile toxins (p>0.5) or between Cryptosporidium spp. and C. difficile culture (p>0.58).

Eight calf PCR ribotypes were identified among 31 C. difficile isolates (Figure). Of these, 7 ribotypes represented by 30 (96.7%) isolates were toxigenic (Table 2). Isolates from 5 ribotypes had the classic tcdC fragment, and ribotypes A11 and F12 had the major type A deletion (≈39 bp deletion) (Table 2). Isolates of ribotype D189 had a tcdC fragment, which is indicative of either a type B or C deletion (≈18 bp).

**Figure:**
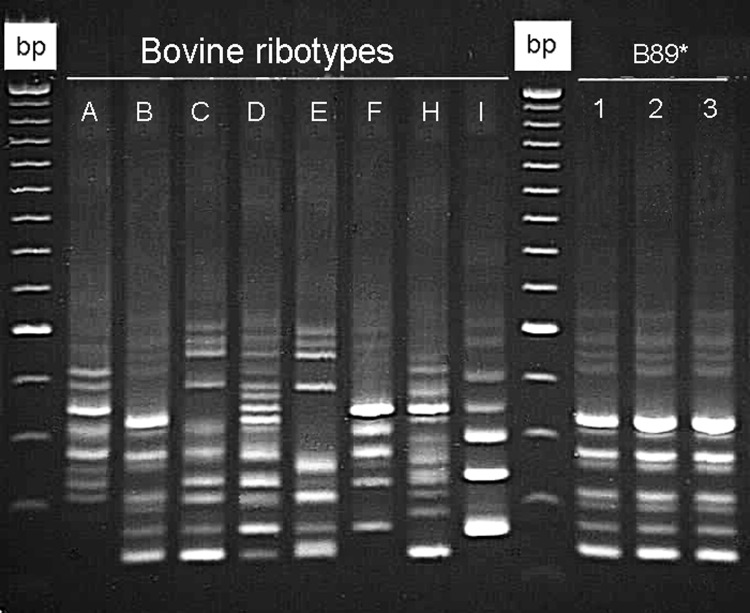
*Clostridium difficile* PCR ribotypes of bovine origin (dairy calves), Ontario, Canada, 2004. *Calf isolate classified as PCR ribotype 017 at the Anaerobe Reference Laboratory, University Hospital of Wales, Cardiff, United Kingdom. Isolates of human (lane 1), calf (lane 2), and canine (lane 3) origin identified in Ontario are indistinguishable. The first and tenth wells contain 100-bp molecular mass markers.

**Table 2:** *Clostridium difficile* PCR ribotypes and toxin genes of 31 isolates obtained from dairy calves in southern Ontario, Canada, 2004

Toxigenic classification†	Calf PCR ribotypes*								Subtotal, no. (%)

	A-11	B-89	C-129	D-189	E-257	F-12	H-75	I-157	

A–B^–^, *cdtB*^–^	–	–	–	–	–	1	–	–	1 (3.2)
A–B^–^, *cdtB*^+^	–	–	–	–	–	2	–	–	2 (6.5)
A+B^+^, *cdtB*^–^	–	–	2	–	4	–	1	1	8 (25.8)
A+B^+^, *cdtB*^+^	7	–	–	4	–	–	–	–	11 (35.5)
A–B^+^, *cdtB*^–^	–	8	–	–	–	–	–	–	8 (25.8)
A–B^+^, *cdtB*^+^	–	1	–	–	–	–	–	–	1 (3.2)
Subtotal, no. (%)	7 (22.6)	9 (29)	2 (6.5)	4 (12.9)	4 (12.9)	3 (9.7)	1 (3.2)	1 (3.2)	31 (100)
Type of *tcdC* deletion‡, (no.)	A (7)	None (9)	None (2)	B (4)	None (4)	A (3)	None (1)	None (1)	
Human PCR ribotypes§	078	017	077	027	014	033	078	NS¶	

*By PCR typing method of Bidet et al. (*22*). †A, toxic gene *tcdA*; B, *tcdB*; – absence or presence of the categorized gene; *cdtB*, gene that codifies CDTb, the binding segment of the binary toxin. ‡None, *tcdC* with no deletions (≈345 bp); A, type A deletion of ≈39 bp; B, nondifferentiated type B or C deletion of ≈18 bp (*11*). §Selected isolates processed at the Anaerobe Reference Laboratory, University Hospital of Wales, Cardiff, UK (*23*). ¶NS, not submitted for analysis.									





The MIC range, MIC_50_, MIC_90_, and the percentage of resistant C. difficile isolates are shown in Table 3. All 30 isolates tested were susceptible to metronidazole and vancomycin. The prevalence of resistance for clindamycin and levofloxacin was similar (73%, 22/30 isolates), but 18 (82%) of the 22 resistant isolates were resistant to both antimicrobial drugs. Calf ribotypes A11 (5/9 isolates), B89 (9/9), C129 (2/2), and D189 (4/4) were overrepresented among the resistant isolates.

**Table 3:** MIC50 and MIC90 range and resistance frequencies of 30 bovine *Clostridium difficile* isolates to 4 antimicrobial drugs by E-test on Muller-Hinton agar after 48 h of incubation*

Drug	MIC_50_, μg/mL	MIC_90_, μg/mL	Range, μg/mL	Resistant isolates, % (no. resistant/no. tested), MIC (μg/mL)	Overrepresented PCR ribotypes (no.)

Vancomycin	0.5	0.75	0.25–1.5	0	0
Metronidazole	0.38	0.75	0.125–2.0	0	0
Levofloxacin	32	32	4 to >32	73 (22/30), >32	B89 (9/9), C129 (2/2), D189 (4/4), other 3 ribotypes (7 isolates)
Clindamycin	16.0	>256	6 to >256	73 (22/30), >12; 37 (11/30), >256	B89 (9/9), C129 (2/2)


*The breakpoints used were vancomycin susceptible, <4.0 μg/mL; vancomycin resistant, >32.0 μg/mL; metronidazole susceptible, <8.0 μg/mL; metronidazole resistant, >32.0 μg/mL; clindamycin susceptible, <2.0 μg/mL; clindamycin resistant >8.0 μg/mL; levofloxacin susceptible, <2.0 μg/mL; levofloxacin resistant, >8.0 μg/mL.

Comparison of the 8 calf PCR ribotypes with a local collection of 25 ribotypes of C. difficile isolated from humans showed that 3 calf ribotypes representing 17 (54.8%) of 31 isolates were indistinguishable (calf ribotypes B89 and D189) or similar (calf ribotype C129) to ribotypes associated with CDAD in humans in Ontario and Quebec (Figure). Ribotype B89, a strain that produces toxin B but not toxin A, was indistinguishable from a strain obtained from patients during a nosocomial outbreak of CDAD in Manitoba, Canada (Figure) (*28*). When compared with a collection of canine isolates from southern Ontario (*29*), this ribotype was also identified in healthy dogs (Figure). Isolates B89 and D89 were not clustered; they were isolated from farms distributed across the studied region with ≈500 km between the most distant ones. Comparison of 7/8 calf ribotypes (representing 30/31 isolates) with a C. difficile PCR ribotype library at the Anaerobe Reference Laboratory, University Hospital of Wales, Cardiff, United Kingdom, that contained >160 C. difficile ribotypes showed that all bovine ribotypes have been identified in humans (Table 2). Toxinotyping of isolates from calf ribotypes B89/ARL-UK PCR ribotype 017 and D189/ARL-UK PCR type 027 indicated that they were toxinotypes VIII and III, respectively. Other calf ribotypes were not toxinotyped.

## Discussion

This study has demonstrated that shedding of C. difficile is common in dairy calves in Ontario regardless whether they have enteric disease. The overall prevalence of shedding (11.2%) was similar to that previously reported (*17*). However, that shedding of C. difficile was more common in control animals was surprising, particularly because 96.7% of the isolates were toxigenic. The reason for this finding is unclear, and natural and methodologic reasons should be considered. Whether the isolation method used in this study resulted in identification bias in favor of 1 of the groups (i.e., control animals) is not known. Pretreatment of fecal samples with ethanol has been shown to facilitate the recovery of C. difficile in asymptomatic humans (*18*). However, how this method would work in calves with and without diarrhea is unknown. The dilutional effect of watery stools could have prevented C. difficile from being isolated from calves with diarrhea, or C. difficile may not be a primary pathogen in calves. In addition, the concentration of C. difficile in the intestinal tract may not correlate with the concentration of spores in feces. Since quantitative culture was not performed in this study, conclusions cannot be made.

The pathophysiology and epidemiology of C. difficile are not completely understood in humans, and some studies have reported that asymptomatic colonization with C. difficile may have a protective effect against CDAD (*30*). In humans, 50%–80% of asymptomatic infants may be colonized with toxigenic C. difficile and have its toxins in their feces (*31*). C. difficile has been reported to affect neonatal foals and piglets (*7**,**8*).

Detection of toxins A and B in feces of humans with diarrhea is considered diagnostic for CDAD (*31**,**32*). The positive association between fecal C. difficile toxins and calf diarrhea found in our study indicates that C. difficile might be a pathogen in calves. However, the clinical relevance of this association is uncertain because it is based on the assumption that the ELISA used has acceptable sensitivity and specificity in calves. The validity of this ELISA has not been reported for most animal species, including cattle. For humans and piglets, adequate sensitivities and specificities for this ELISA (65%–95% and 95%–100%, respectively) (*20**,**32**,**33*) contrast with recently reported suboptimal performance for canine feces (*34*). With an apparent interspecies variability of the ELISA, validation of this test for bovine feces is required before conclusions regarding causal associations can be made.

The finding that calves were more likely to have detectable levels of C. difficile toxins in their feces early in life is consistent with findings of a previous study (*17*). The reason for this is unclear, although C. difficile may be better able to colonize, proliferate, and produce toxins in younger animals with less developed intestinal microflora. In other animal species and humans, administration of antimicrobial drugs is considered a predisposing factor for development of CDAD (*3**,**7**,**35**,**36*). No statistical associations were identified in this regard at the calf level because questionnaires were designed to explore farm practices.

Molecular analyses showed that a relevant proportion of the C. difficile isolates (9/31) had tcdB genes but not tcdA genes (A–B^+^). These variant isolates are uncommon in humans but have been reported in association with outbreaks of CDAD (*2**,**36*). In a previous study in calves, no A–B^+^ isolates were identified (*17*). This discrepancy could be due to potential differences between the 2 study populations.

In our study, the 9 calf A–B^+^ isolates and a control strain were classified as ribotype pattern B89 type 017 (Figure). This ribotype has been reported in outbreaks of CDAD in humans in various countries (*2**,**28**,**36*), including the Canadian provinces of Ontario, Quebec, and Manitoba, from which the human control strain was obtained (*28*). Toxinotyping (type VIII) and tcdC analysis (classic gene) of these 9 calf isolates supported their similarity to human strains. The epidemiologic explanation for the presence of this human epidemic strain in calves and in healthy dogs (*29*) is uncertain, but this finding raises the concern of potential animal-to-human transmission and vice versa. No isolates of bovine origin were available for additional retrospective comparisons.

The second major calf ribotype common to humans in Ontario and Quebec was D189/PCR ribotype 027 (positive for tcdA, tcdB and cdtB, type B tcdC deletion, and toxinotype III). Molecular characteristics of this ribotype indicate that it is a hypertoxin-producing ribotype recently reported as a cause of serious outbreaks of disease in humans in North America and Europe (*1*). In Quebec, Canada, C. difficile type 027 was isolated during an outbreak from 67% of persons with hospital-acquired CDAD and 37% of persons with community-acquired CDAD (*1*). The pathogenicity of this ribotype is believed to be associated with a high production of toxins A and B in vitro, and with fluoroquinolone resistance (*3**,**4*).

The 4 calf isolates of PCR D189/ribotype 027 identified in our study were not geographically clustered. This result and the recent finding of this strain in a dog in Ontario indicate that this C. difficile ribotype may be widely disseminated in the community in different animal species (*37*). The public health consequences of this are unclear and require further study. Whether cattle could play a role in dissemination of this strain through direct contact, environmental contamination, or the food chain should be determined. Although C. difficile is not considered a foodborne pathogen, it has been identified in raw meat intended for pet consumption (*38*) and in retail meat from grocery stores in Ontario (A. Rodriguez-Palacios et al., unpub. data).

Results of antimicrobial drug susceptibility tests for metronidazole, vancomycin, and clindamycin are consistent with those of previous reports in humans, in which antimicrobial susceptibility of C. difficile strains to metronidazole and vancomycin was ≈100% and antimicrobial resistance to clindamycin was ≈70%–80% (*2**,**26**,**35*). Most isolates (73%) were resistant to levofloxacin, which is not administered to cattle. Antimicrobial drug resistance to fluoroquinolones has been described in C. difficile PCR ribotype 027 as a major risk factor for development of CDAD (*4**,**5*). The development of fluoroquinolone resistance in human-derived strains has been hypothesized to result from increased use of these antimicrobial drugs, which has also been associated with a higher risk for CDAD in hospitals (*3**,**5*).

Use of fluoroquinolones was not voluntarily reported for any of the farms or calves in this study, and levofloxacin resistance cannot be extrapolated to other fluoroquinolones (*39*). In Canada, fluoroquinolones are not approved for use in dairy cattle or veal calves. Fluoroquinolones have not been approved for veterinary use in any food-producing animals in Canada until recently, when a commercial enrofloxacin product was approved only for use in beef cattle with unresponsive respiratory disease (*39*). As part of a Canadian surveillance program, Health Canada, through the Canadian Integrated Program for Antimicrobial Resistance Surveillance, has monitored fluoroquinolone resistance in strains of Escherichia coli and Salmonella spp. from beef cattle since 2001–2002. According to the Canadian Integrated Program for Antimicrobial Resistance Surveillance 2002 and 2003 reports, no resistance to fluoroquinolones has been observed (*40*). Thus, the source of fluoroquinolone resistance in calf-derived C. difficile isolates in our study is uncertain and is not substantiated on the hypothesis of excessive use of fluoroquinolones, i.e., enrofloxacin, in cattle. Whether this resistance has any epidemiologic association with companion animals (i.e., dogs) or humans for which fluoroquinolones have been approved for many years remains unknown.

The results of our study indicate that C. difficile may play a role in neonatal calf diarrhea, which is a serious concern in the bovine industry. Calf C. difficile isolates that are indistinguishable from human strains and have fluoroquinolone resistance and tcdC deletions also raise the possibility of interspecies transmission. Although this study did not confirm that infection with C. difficile is zoonotically transmitted, further study is indicated to evaluate this possibility. Investigations of recent changes in the epidemiology of CDAD and identification of new pathogenic genotypes should also involve concurrent evaluation of animal reservoirs or origins. Validation studies are also required to assess culture protocols and immunoassay tests for identification of C. difficile and its toxins in cattle feces.
